# Supplementary material for: Decapping activators Edc3 and Scd6 act redundantly with Dhh1 in post-transcriptional repression of starvation-induced pathways
Source: eLife. 2025 Nov 25;13:RP102287. doi: 10.7554/eLife.102287 (PMC12646578; doi:10.7554/eLife.102287)
Supplement: Figure 3—source data 3. [file elife-102287-fig3-data3.zip › Fig. 3F-source data 1. PPT file containing original blots indicating relevant bands_10-27-25.pptx]

## Slide 1
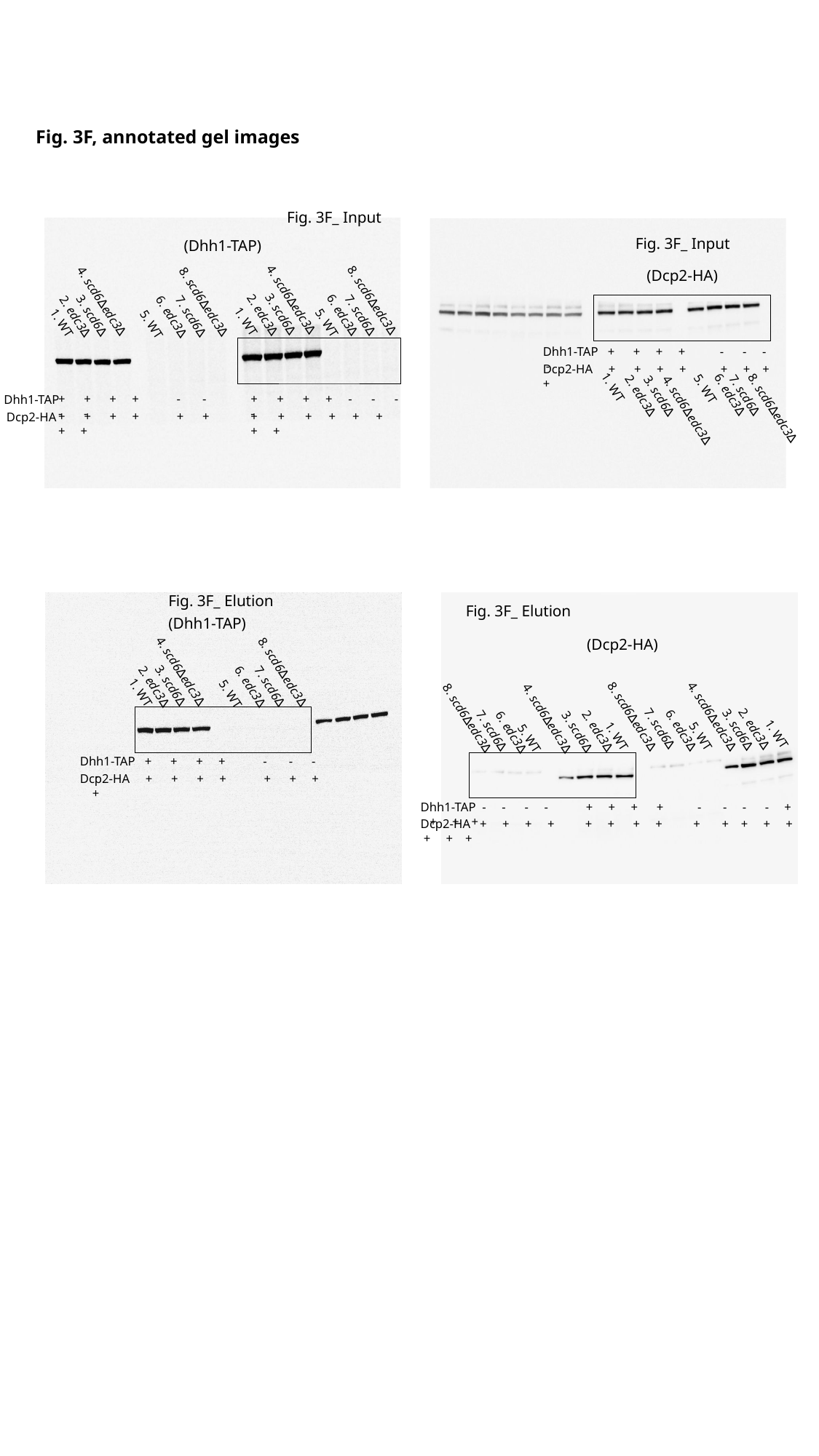

Fig. 3F, annotated gel images
Fig. 3F_ Input
(Dhh1-TAP)
4. scd6∆edc3∆
8. scd6∆edc3∆
4. scd6∆edc3∆
8. scd6∆edc3∆
3. scd6∆
2. edc3∆
7. scd6∆
6. edc3∆
3. scd6∆
2. edc3∆
7. scd6∆
6. edc3∆
1. WT
5. WT
1. WT
5. WT
+ + + + - - - -
+ + + + - - - -
Dhh1-TAP
+ + + + + + + +
+ + + + + + + +
Dcp2-HA
Fig. 3F_ Input
(Dcp2-HA)
Dhh1-TAP + + + + - - - -
Dcp2-HA + + + + + + + +
1. WT
5. WT
6. edc3∆
7. scd6∆
2. edc3∆
3. scd6∆
8. scd6∆edc3∆
4. scd6∆edc3∆
Fig. 3F_ Elution
(Dhh1-TAP)
4. scd6∆edc3∆
8. scd6∆edc3∆
3. scd6∆
2. edc3∆
7. scd6∆
6. edc3∆
1. WT
5. WT
Dhh1-TAP + + + + - - - -
Dcp2-HA + + + + + + + +
Fig. 3F_ Elution
(Dcp2-HA)
8. scd6∆edc3∆
4. scd6∆edc3∆
8. scd6∆edc3∆
4. scd6∆edc3∆
7. scd6∆
2. edc3∆
3. scd6∆
6. edc3∆
7. scd6∆
2. edc3∆
3. scd6∆
6. edc3∆
1. WT
1. WT
5. WT
5. WT
Dhh1-TAP - - - - + + + + - - - - + + + +
Dcp2-HA + + + + + + + + + + + + + + + +
