# Supplementary figures and images for: Decapping activators Edc3 and Scd6 act redundantly with Dhh1 in post-transcriptional repression of starvation-induced pathways
Source: eLife. 2025 Nov 25;13:RP102287. doi: 10.7554/eLife.102287 (PMC12646578; doi:10.7554/eLife.102287)

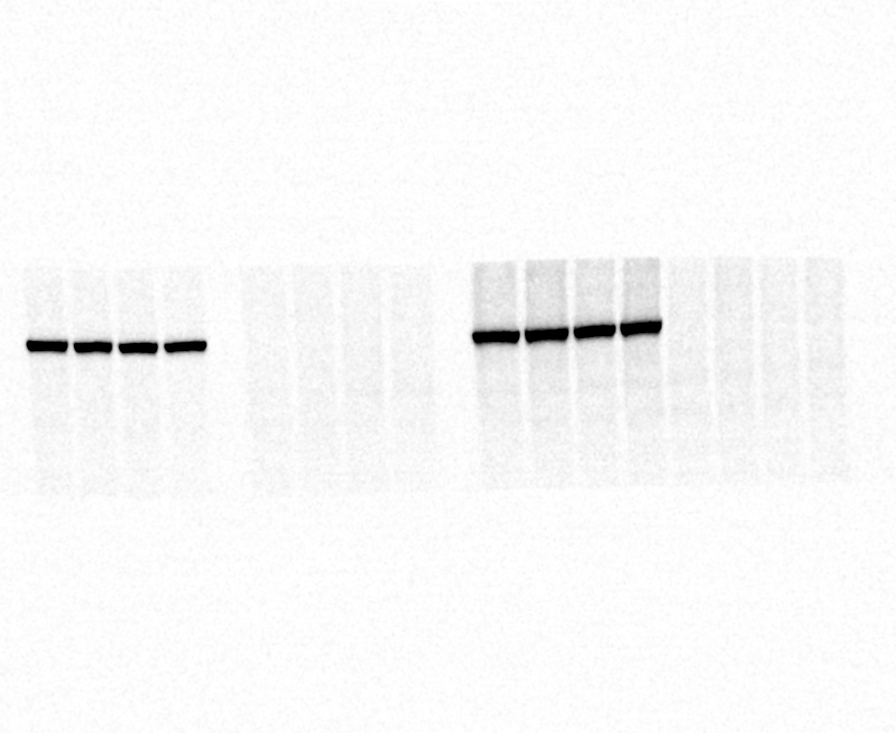

Supplement: Figure 3—source data 4. [file elife-102287-fig3-data4.zip › Fig. 3F-source data 2. original files for western analysis_10-27-25/DHH1-TAP input Fig 3F and Fig3-Fig Supp2 (i) Raw.jpg]

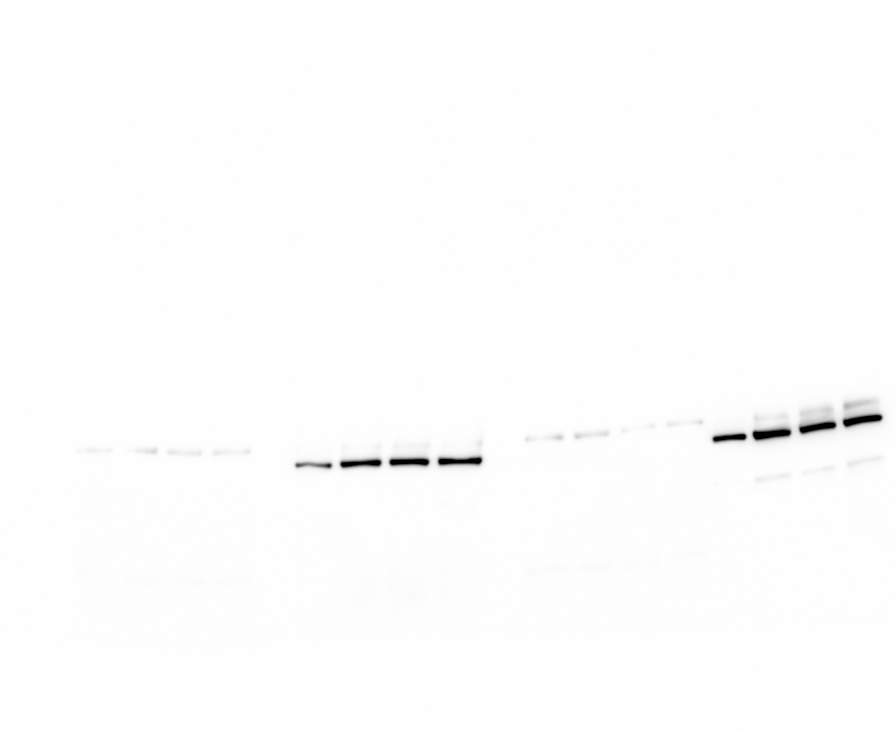

Supplement: Figure 3—source data 4. [file elife-102287-fig3-data4.zip › Fig. 3F-source data 2. original files for western analysis_10-27-25/Dcp2-HA, elution Fig 3F and Fig3-Fig Supp2 (ii) Raw.jpg]

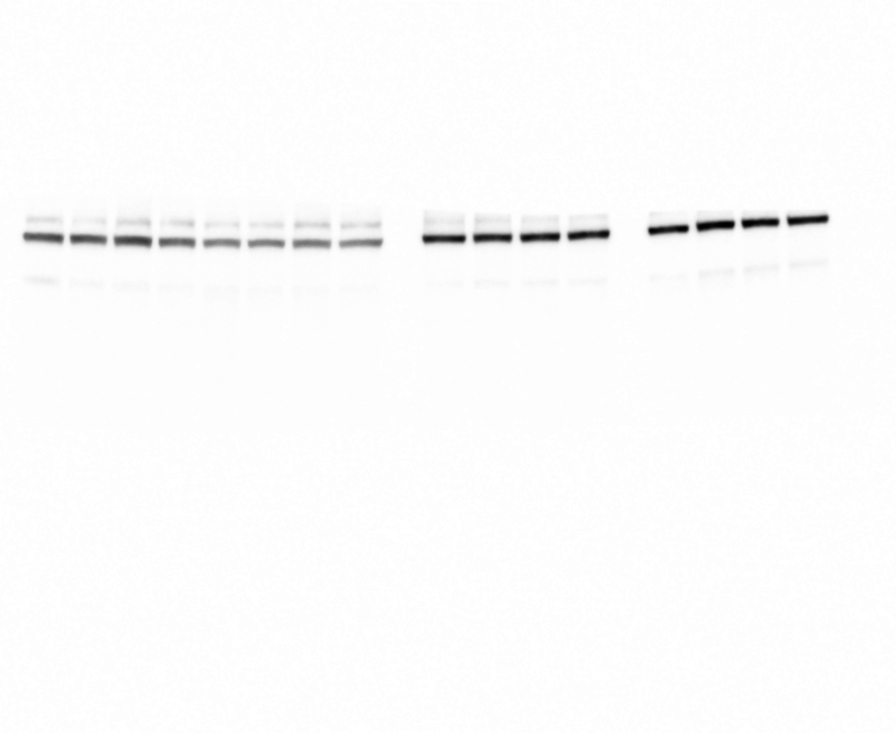

Supplement: Figure 3—source data 4. [file elife-102287-fig3-data4.zip › Fig. 3F-source data 2. original files for western analysis_10-27-25/Dcp2-HA, input Fig 3F Raw.jpg]

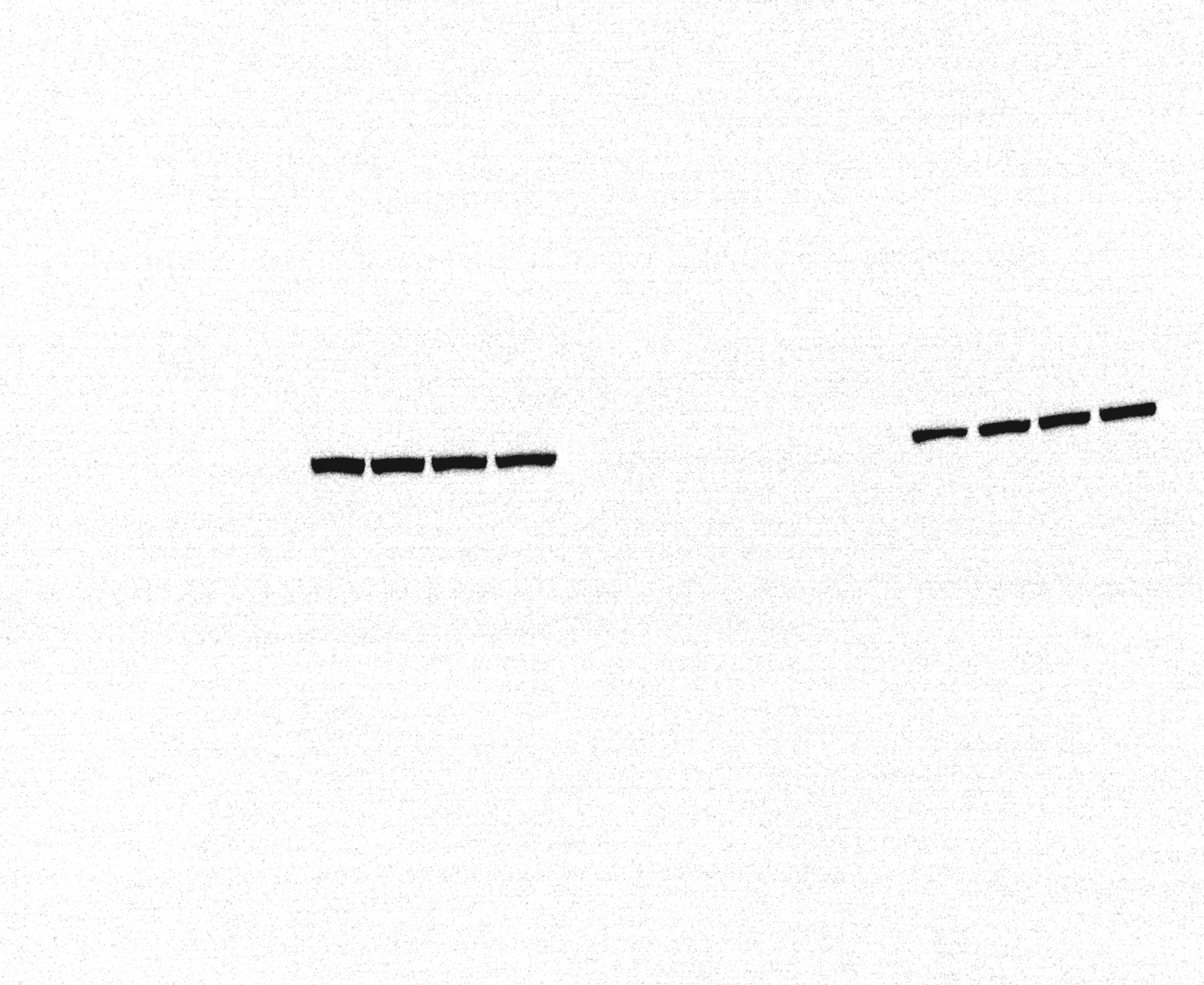

Supplement: Figure 3—source data 4. [file elife-102287-fig3-data4.zip › Fig. 3F-source data 2. original files for western analysis_10-27-25/Dhh1-TAP elution Fig 3F Raw.jpg]

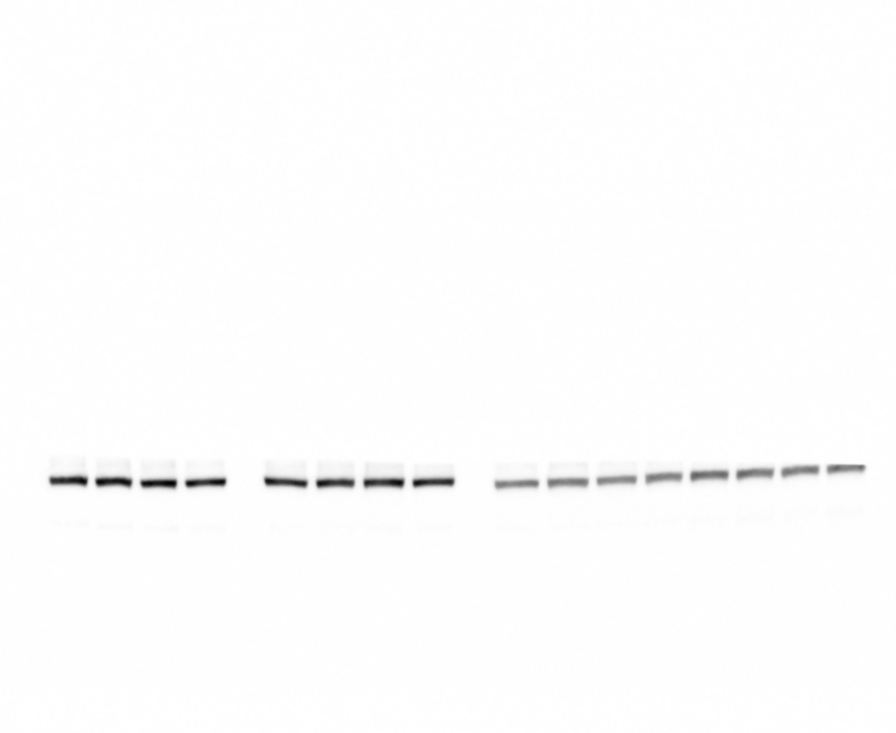

Supplement: Figure 3—figure supplement 2—source data 2. [file elife-102287-fig3-figsupp2-data2.zip › Fig. 3-Figure supplement 2-source data 2. original files for western analysis_10-27-25/Dcp2-HA input Fig3-Fig Supp2 (ii)Raw.jpg]

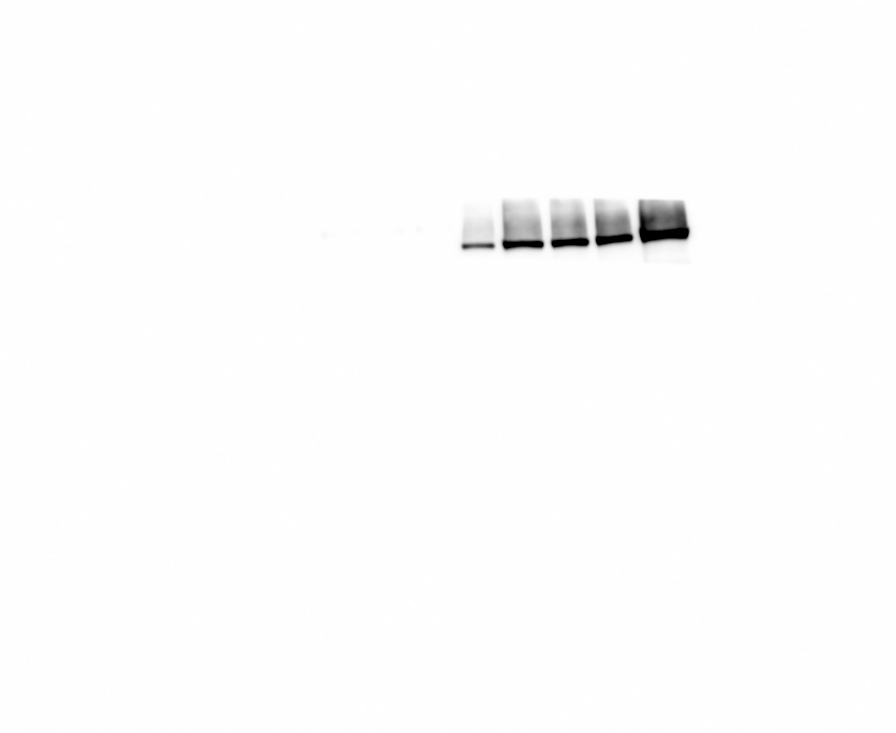

Supplement: Figure 3—figure supplement 2—source data 2. [file elife-102287-fig3-figsupp2-data2.zip › Fig. 3-Figure supplement 2-source data 2. original files for western analysis_10-27-25/Dcp2-HA, elution Fig 3-Fig Supp2 (i) Raw.jpg]

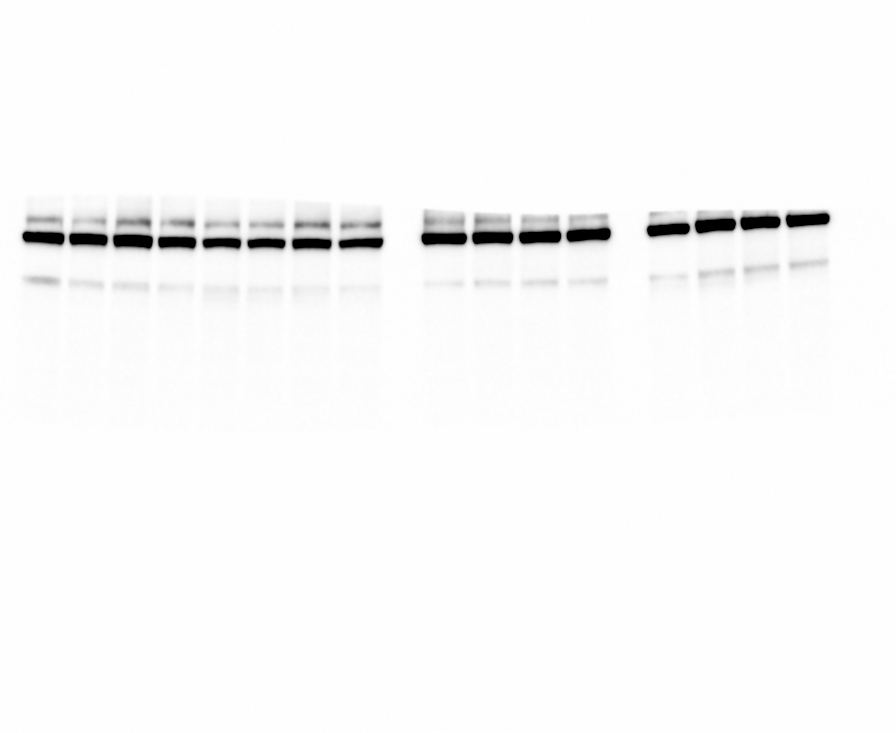

Supplement: Figure 3—figure supplement 2—source data 2. [file elife-102287-fig3-figsupp2-data2.zip › Fig. 3-Figure supplement 2-source data 2. original files for western analysis_10-27-25/Dcp2-HA, input Fig3-Fig Supp2 (i) Raw.jpg]

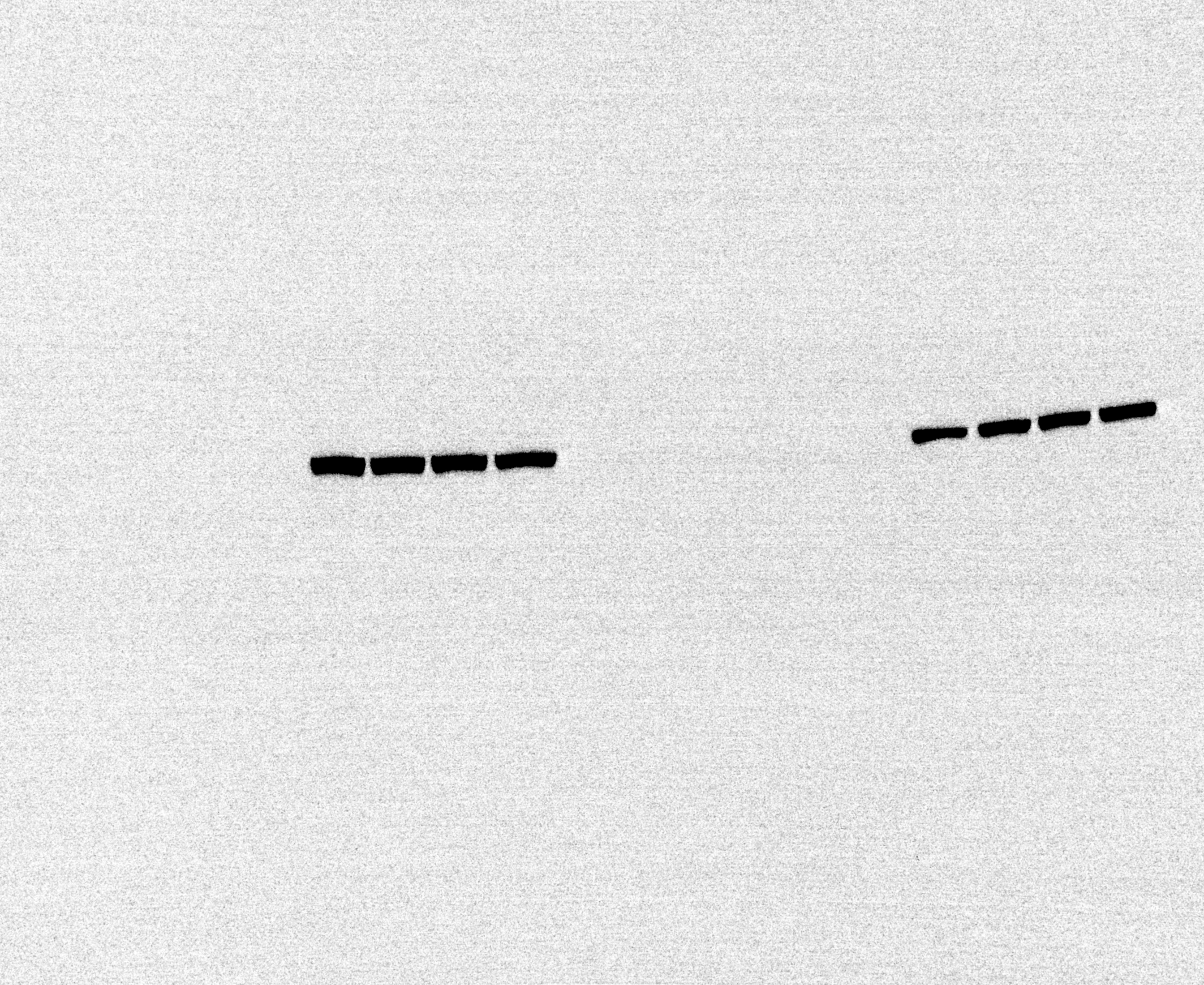

Supplement: Figure 3—figure supplement 2—source data 2. [file elife-102287-fig3-figsupp2-data2.zip › Fig. 3-Figure supplement 2-source data 2. original files for western analysis_10-27-25/Dhh1-TAP elution Fig 3_Fig Supp2 (i) Raw.jpg]

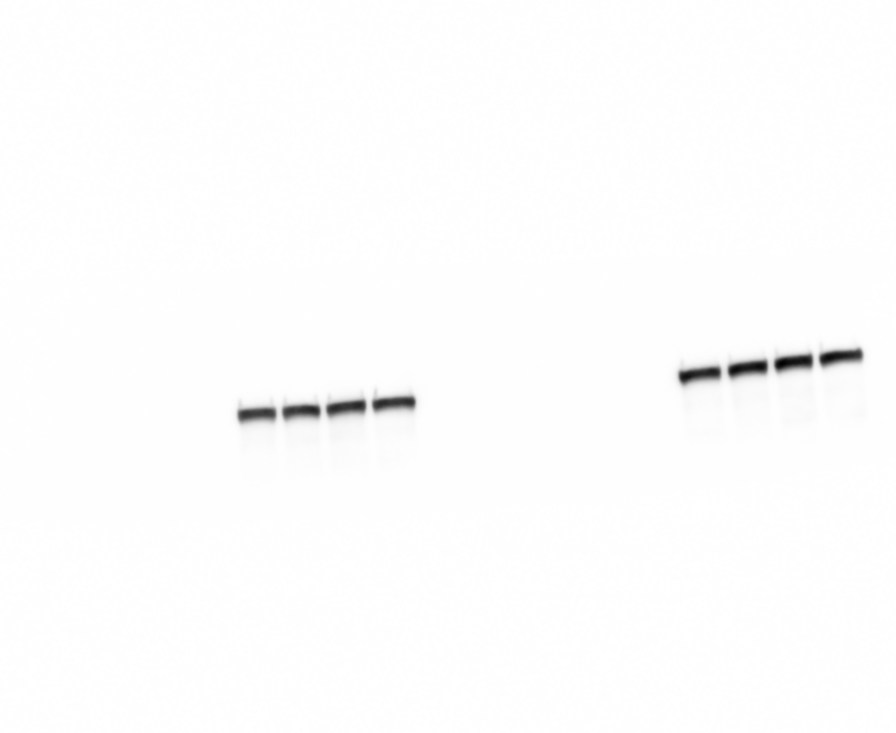

Supplement: Figure 3—figure supplement 2—source data 2. [file elife-102287-fig3-figsupp2-data2.zip › Fig. 3-Figure supplement 2-source data 2. original files for western analysis_10-27-25/Dhh1-TAP input and elution Fig3_Fig Supp2 (ii) Raw.jpg]

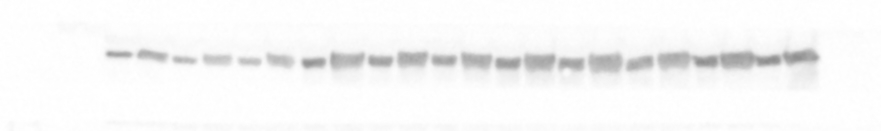

Supplement: Figure 6—source data 3. [file elife-102287-fig6-data3.zip › Fig. 6B-source data 2. original files for western analysis_10-28-25/Anti_Pet10_4.jpg]

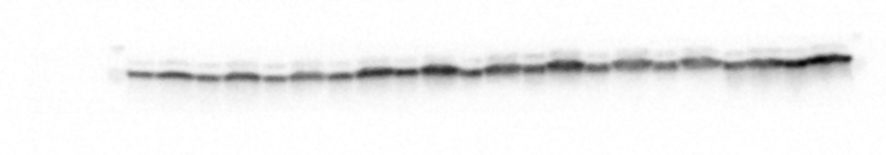

Supplement: Figure 6—source data 3. [file elife-102287-fig6-data3.zip › Fig. 6B-source data 2. original files for western analysis_10-28-25/Anti_Qcr8_11reprobe.jpg]

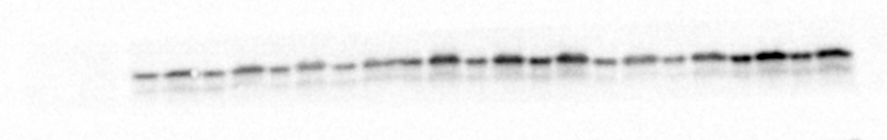

Supplement: Figure 6—source data 3. [file elife-102287-fig6-data3.zip › Fig. 6B-source data 2. original files for western analysis_10-28-25/Anti_Sdh4_16_reprobe.jpg]

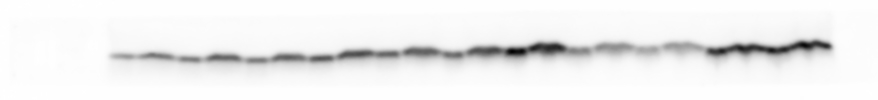

Supplement: Figure 6—source data 3. [file elife-102287-fig6-data3.zip › Fig. 6B-source data 2. original files for western analysis_10-28-25/Anti_Atp20_11.jpg]

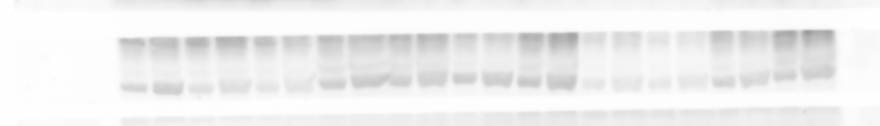

Supplement: Figure 6—source data 3. [file elife-102287-fig6-data3.zip › Fig. 6B-source data 2. original files for western analysis_10-28-25/Anti_Cit2_14.jpg]

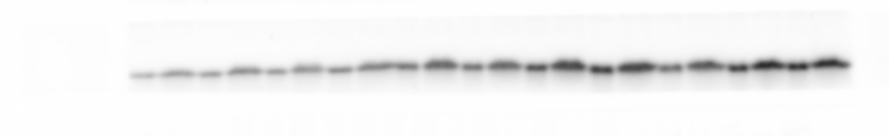

Supplement: Figure 6—source data 3. [file elife-102287-fig6-data3.zip › Fig. 6B-source data 2. original files for western analysis_10-28-25/Anti_Cox14_16.jpg]

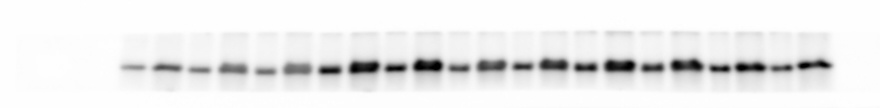

Supplement: Figure 6—source data 3. [file elife-102287-fig6-data3.zip › Fig. 6B-source data 2. original files for western analysis_10-28-25/Anti_Cox20_10.jpg]

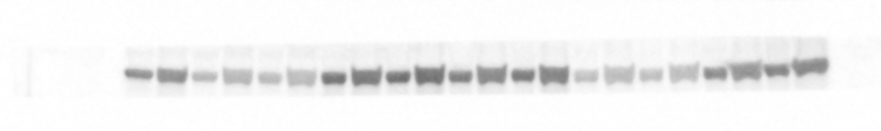

Supplement: Figure 6—source data 3. [file elife-102287-fig6-data3.zip › Fig. 6B-source data 2. original files for western analysis_10-28-25/Anti_Cyb2_8.jpg]

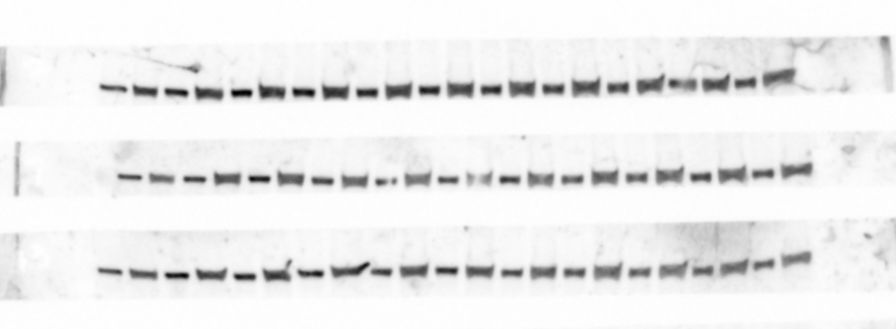

Supplement: Figure 6—source data 3. [file elife-102287-fig6-data3.zip › Fig. 6B-source data 2. original files for western analysis_10-28-25/Anti_Gcd6_2_7_13.jpg]

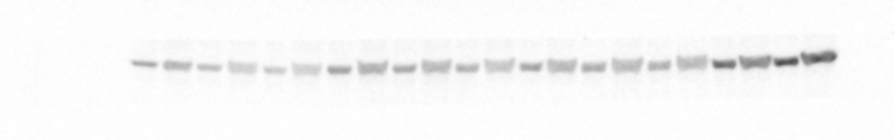

Supplement: Figure 6—source data 3. [file elife-102287-fig6-data3.zip › Fig. 6B-source data 2. original files for western analysis_10-28-25/Anti_Idh1_9.jpg]

## Slide 1
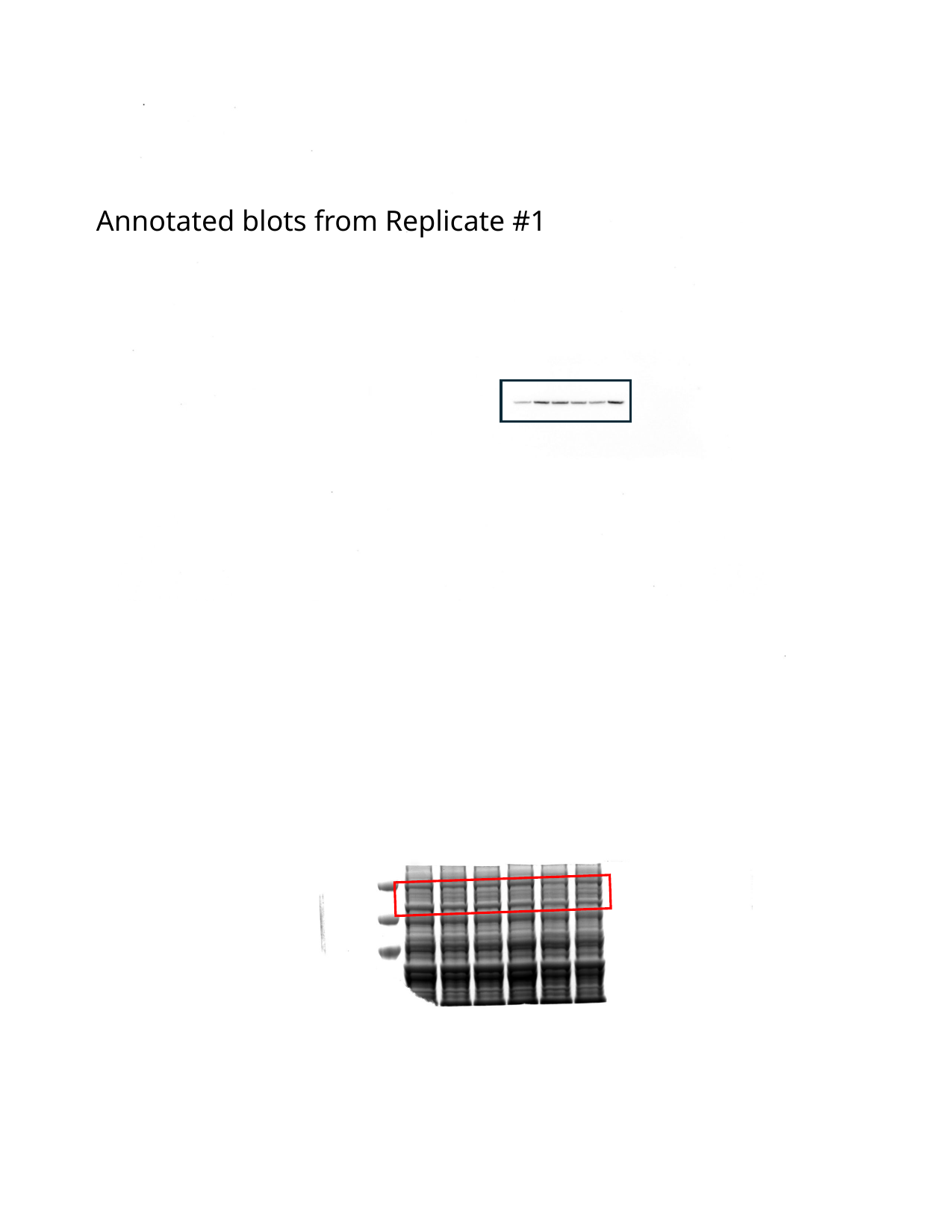

Annotated blots from Replicate #1

Supplement: Figure 6—source data 4. [file elife-102287-fig6-data4.zip › Fig. 6D-source data 1. PPT file containing original blots indicating relevant bands_10-27-25.pptx]

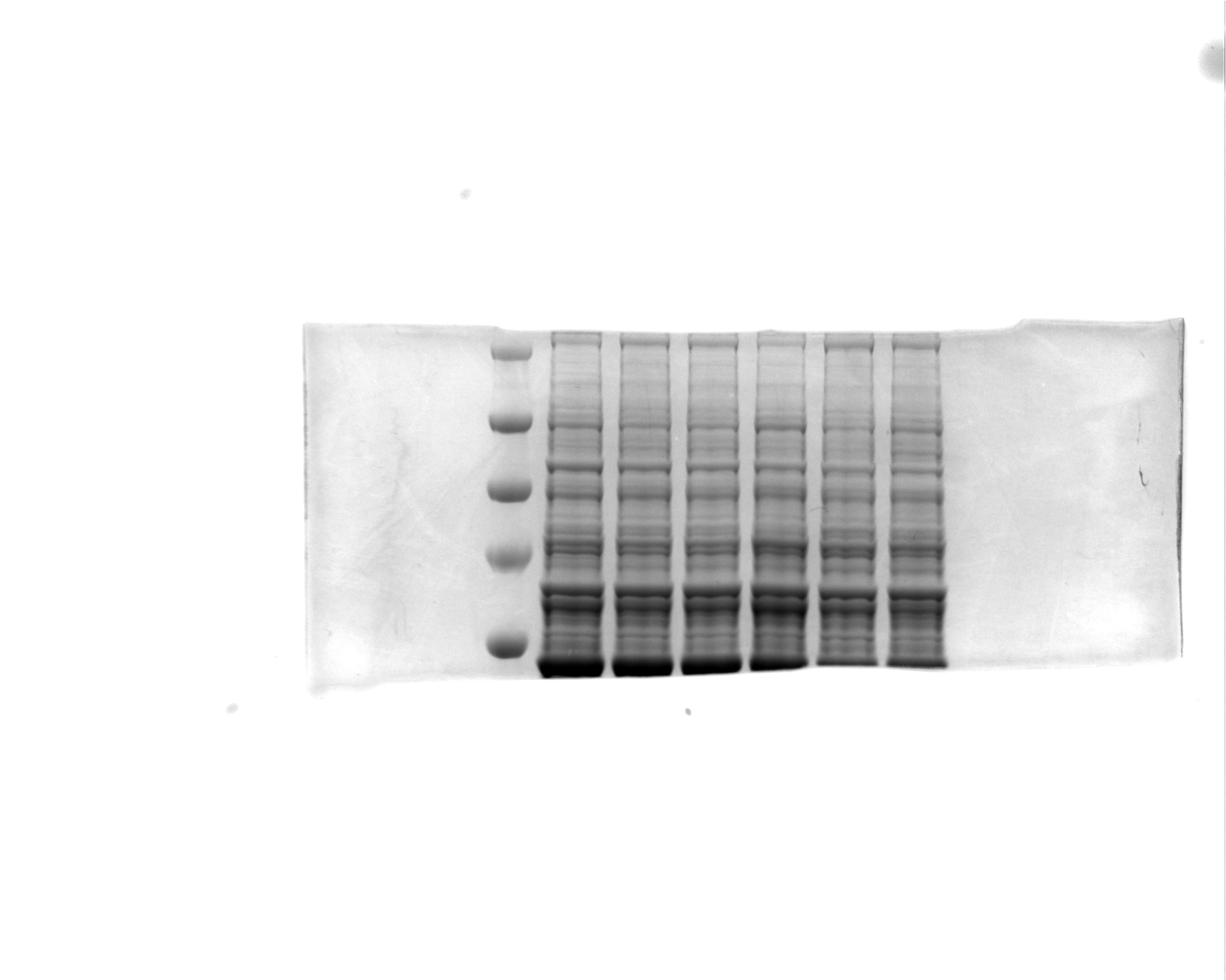

Supplement: Figure 6—source data 5. [file elife-102287-fig6-data5.zip › Fig. 6D-source data 2. original files for western analysis_10-27-25/rep 3/PROTEIN_GEL_03062024_172143.tif]

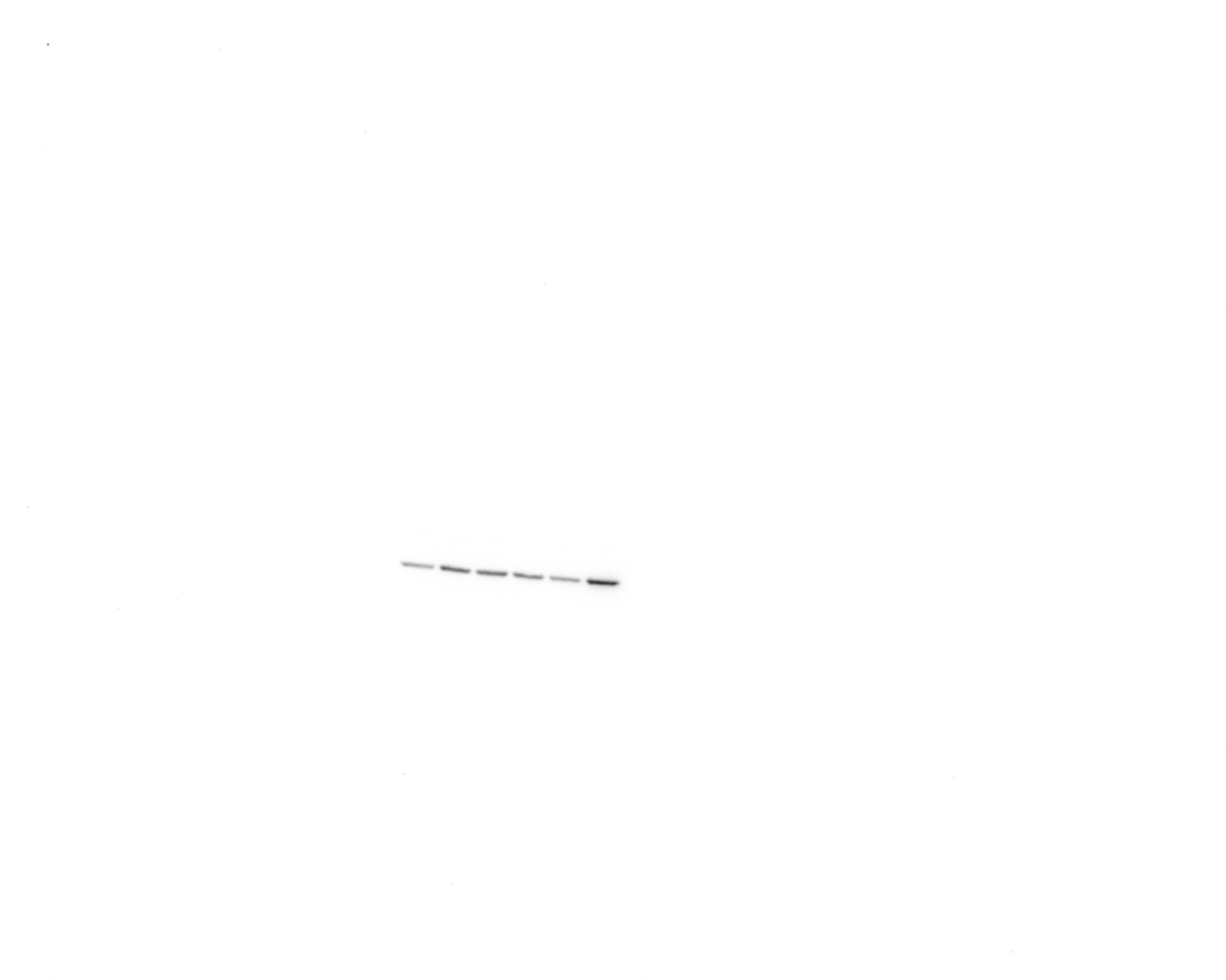

Supplement: Figure 6—source data 5. [file elife-102287-fig6-data5.zip › Fig. 6D-source data 2. original files for western analysis_10-27-25/rep 3/CHEMI_03052024_144151.tif]

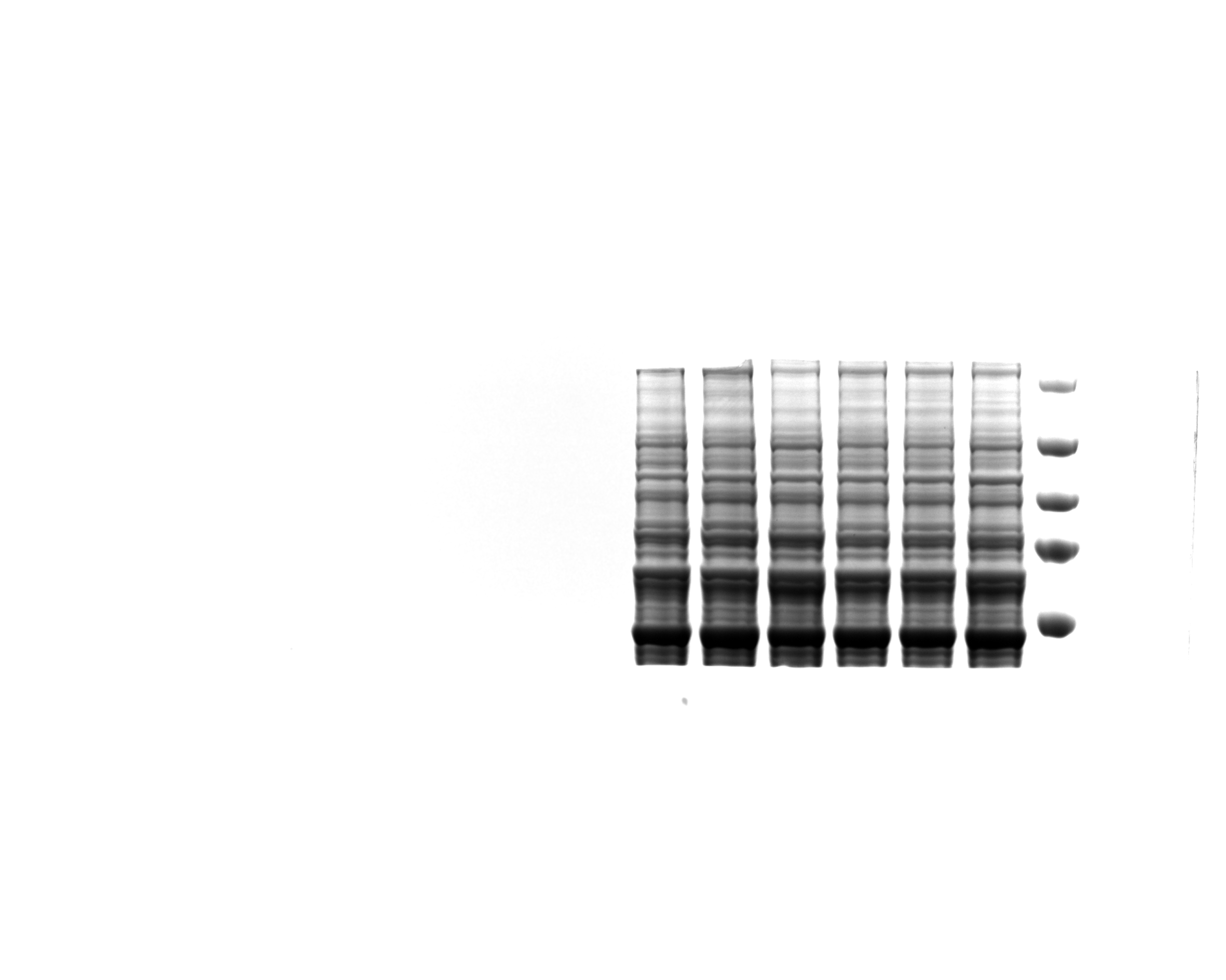

Supplement: Figure 6—source data 5. [file elife-102287-fig6-data5.zip › Fig. 6D-source data 2. original files for western analysis_10-27-25/rep 2/PROTEIN_GEL_03062024_171847.tif]

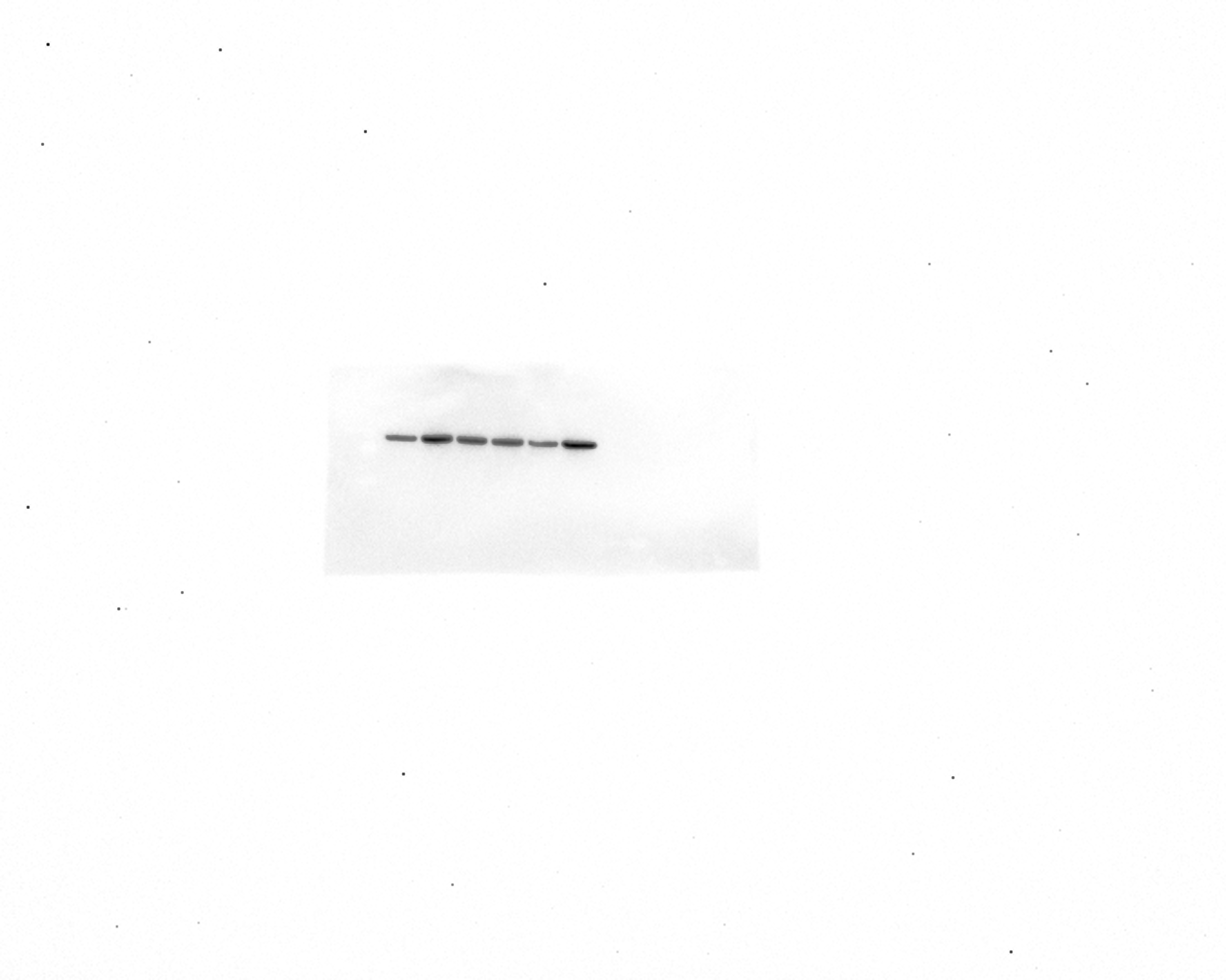

Supplement: Figure 6—source data 5. [file elife-102287-fig6-data5.zip › Fig. 6D-source data 2. original files for western analysis_10-27-25/rep 2/CHEMI_03022024_194322.tif]

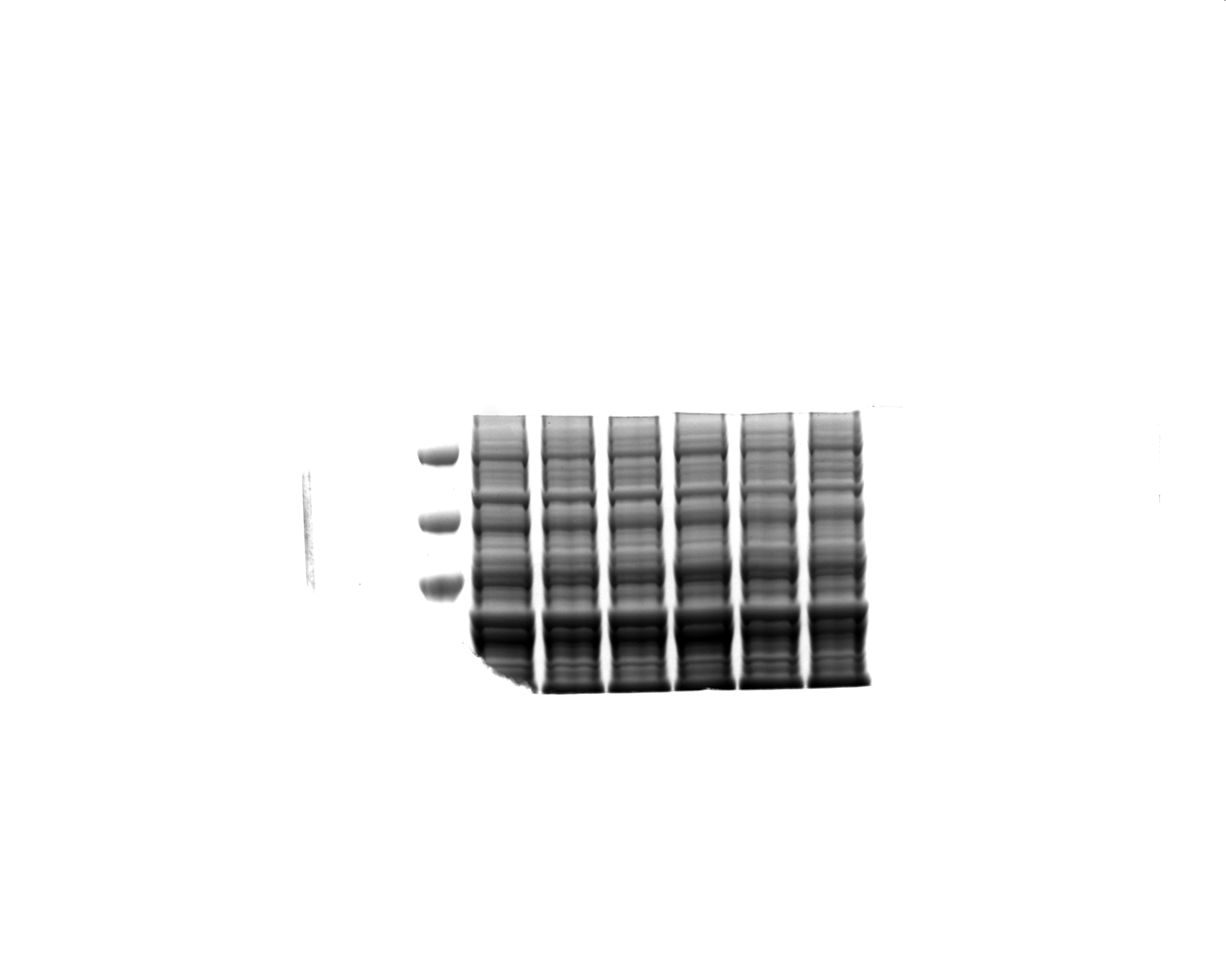

Supplement: Figure 6—source data 5. [file elife-102287-fig6-data5.zip › Fig. 6D-source data 2. original files for western analysis_10-27-25/rep 1/2024_02_29_181302.tif]

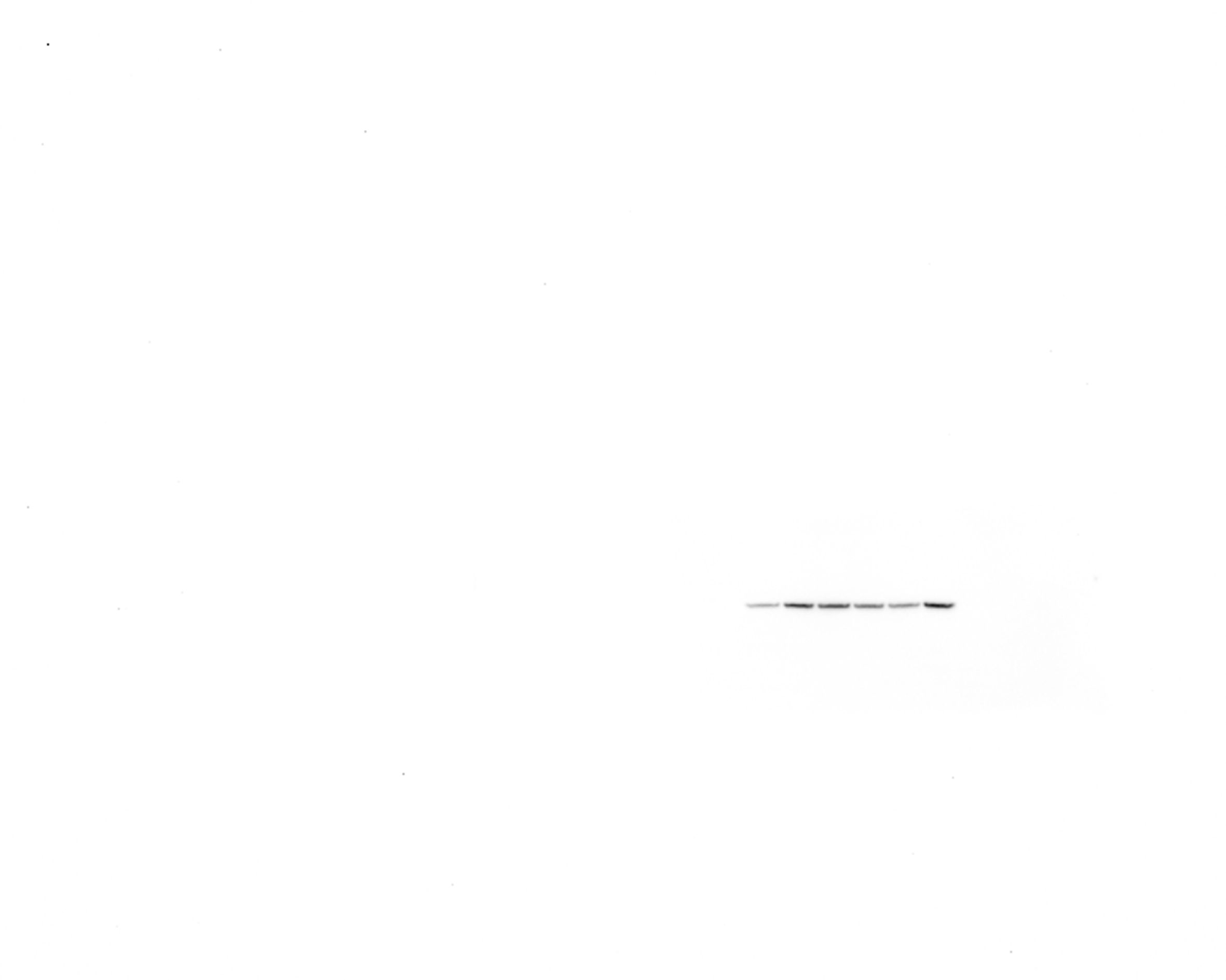

Supplement: Figure 6—source data 5. [file elife-102287-fig6-data5.zip › Fig. 6D-source data 2. original files for western analysis_10-27-25/rep 1/CHEMI_02282024_185917.tif]
