# Supplementary material for: Decapping activators Edc3 and Scd6 act redundantly with Dhh1 in post-transcriptional repression of starvation-induced pathways
Source: eLife. 2025 Nov 25;13:RP102287. doi: 10.7554/eLife.102287 (PMC12646578; doi:10.7554/eLife.102287)
Supplement: Figure 3—figure supplement 2—source data 1. [file elife-102287-fig3-figsupp2-data1.zip › Fig. 3-Figure Supplement 2-source data 1. PPT file containing original blots indicating relevant bands_10-27-25.pptx]

## Slide 1
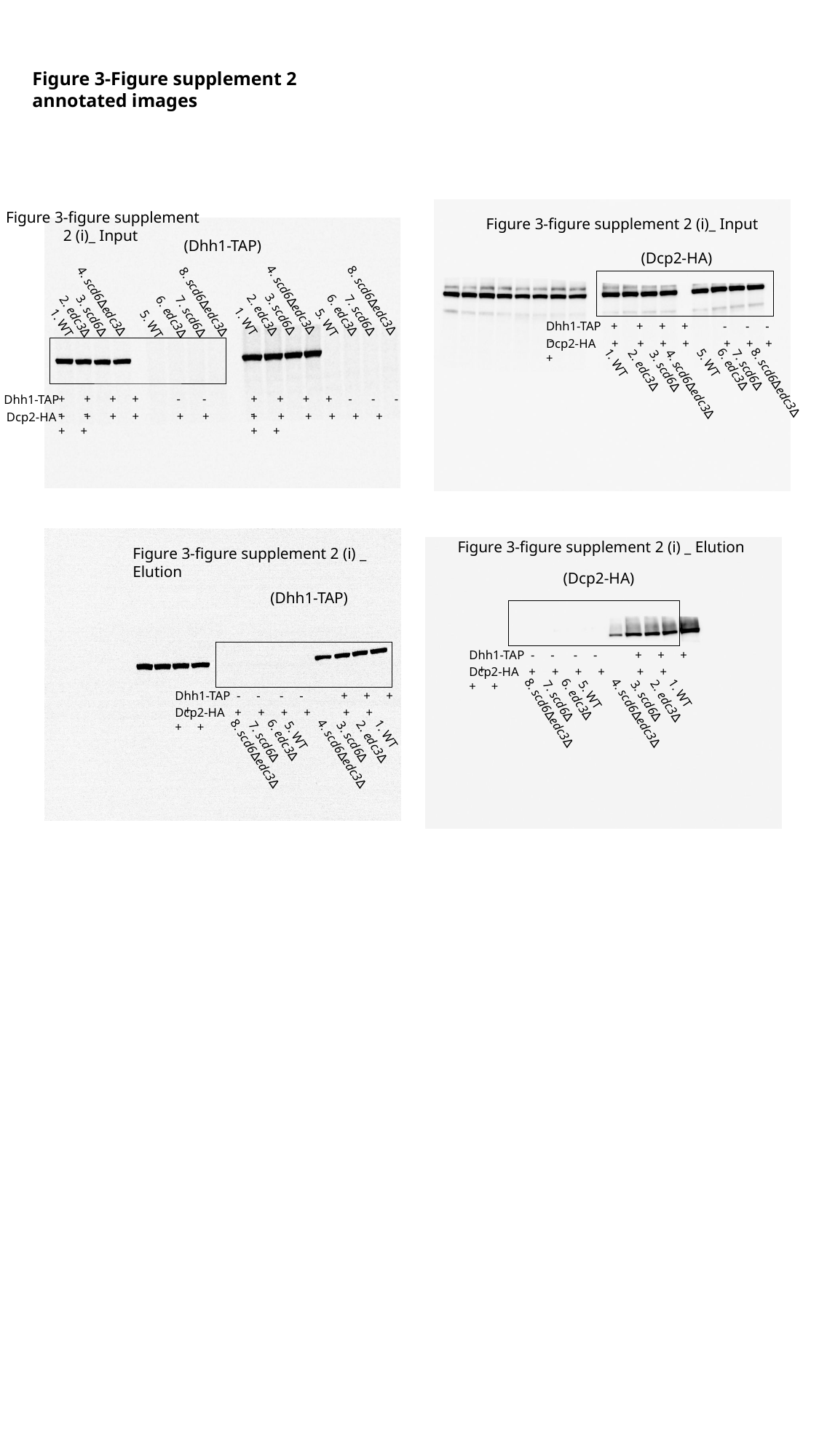

Figure 3-Figure supplement 2 annotated images
Figure 3-figure supplement 2 (i)_ Input
(Dcp2-HA)
Dhh1-TAP + + + + - - - -
Dcp2-HA + + + + + + + +
1. WT
5. WT
6. edc3∆
7. scd6∆
2. edc3∆
3. scd6∆
8. scd6∆edc3∆
4. scd6∆edc3∆
Figure 3-figure supplement 2 (i)_ Input
(Dhh1-TAP)
4. scd6∆edc3∆
8. scd6∆edc3∆
4. scd6∆edc3∆
8. scd6∆edc3∆
3. scd6∆
2. edc3∆
7. scd6∆
6. edc3∆
3. scd6∆
2. edc3∆
7. scd6∆
6. edc3∆
1. WT
5. WT
1. WT
5. WT
+ + + + - - - -
+ + + + - - - -
Dhh1-TAP
+ + + + + + + +
+ + + + + + + +
Dcp2-HA
Figure 3-figure supplement 2 (i) _ Elution
(Dhh1-TAP)
Dhh1-TAP - - - - + + + +
Dcp2-HA + + + + + + + +
1. WT
5. WT
6. edc3∆
7. scd6∆
3. scd6∆
2. edc3∆
8. scd6∆edc3∆
4. scd6∆edc3∆
Figure 3-figure supplement 2 (i) _ Elution
(Dcp2-HA)
Dhh1-TAP - - - - + + + +
Dcp2-HA + + + + + + + +
1. WT
5. WT
6. edc3∆
7. scd6∆
3. scd6∆
2. edc3∆
8. scd6∆edc3∆
4. scd6∆edc3∆

## Slide 2
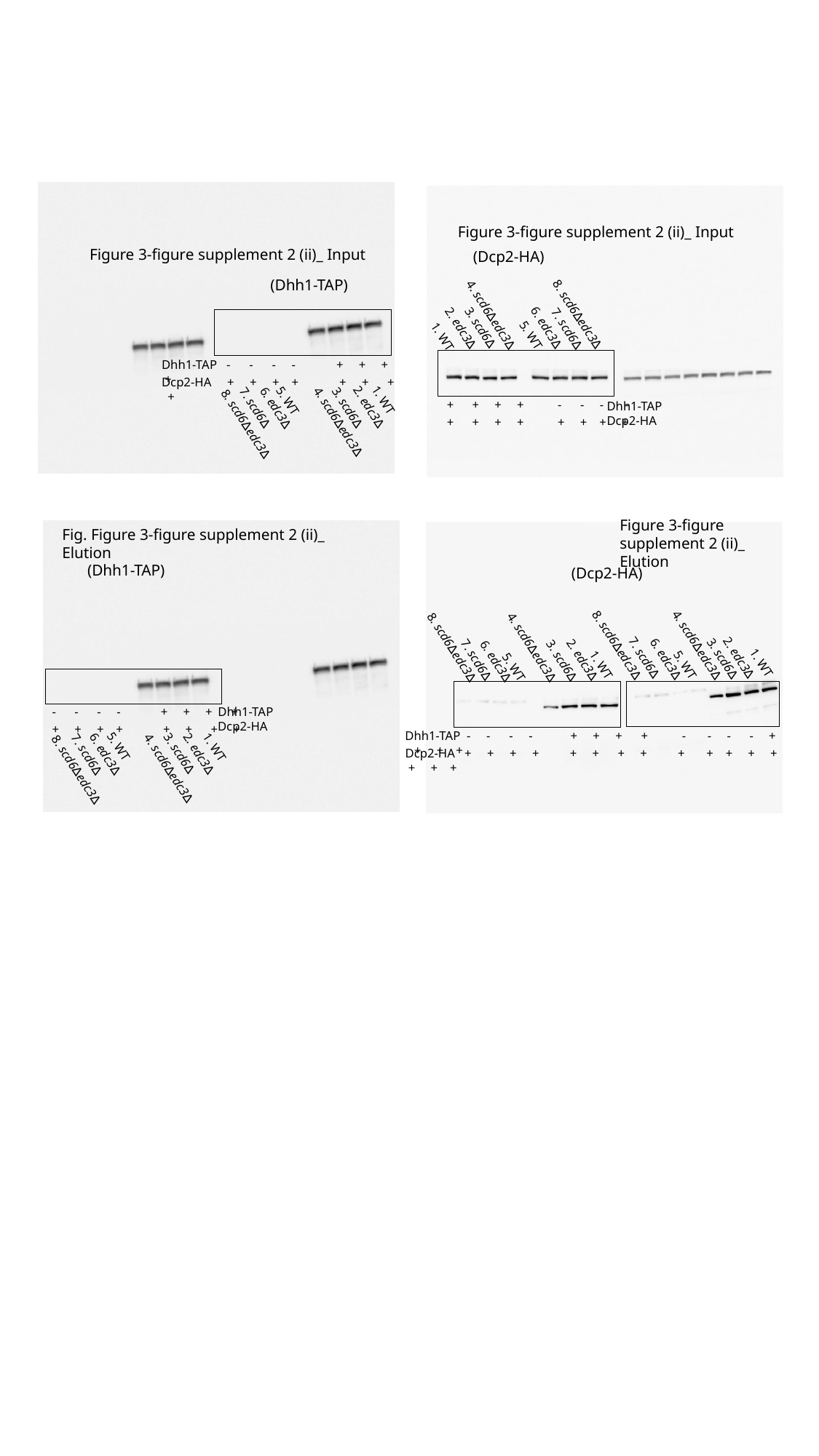

Figure 3-figure supplement 2 (ii)_ Input
(Dhh1-TAP)
Dhh1-TAP - - - - + + + +
Dcp2-HA + + + + + + + +
1. WT
5. WT
2. edc3∆
3. scd6∆
7. scd6∆
6. edc3∆
4. scd6∆edc3∆
8. scd6∆edc3∆
Figure 3-figure supplement 2 (ii)_ Input
(Dcp2-HA)
8. scd6∆edc3∆
4. scd6∆edc3∆
6. edc3∆
3. scd6∆
2. edc3∆
7. scd6∆
5. WT
1. WT
+ + + + - - - -
Dhh1-TAP
Dcp2-HA
+ + + + + + + +
Figure 3-figure supplement 2 (ii)_ Elution
(Dcp2-HA)
8. scd6∆edc3∆
4. scd6∆edc3∆
8. scd6∆edc3∆
4. scd6∆edc3∆
7. scd6∆
2. edc3∆
3. scd6∆
6. edc3∆
7. scd6∆
2. edc3∆
3. scd6∆
6. edc3∆
1. WT
1. WT
5. WT
5. WT
Dhh1-TAP - - - - + + + + - - - - + + + +
Dcp2-HA + + + + + + + + + + + + + + + +
Fig. Figure 3-figure supplement 2 (ii)_ Elution
(Dhh1-TAP)
- - - - + + + +
Dhh1-TAP
Dcp2-HA
+ + + + + + + +
5. WT
1. WT
3. scd6∆
2. edc3∆
6. edc3∆
7. scd6∆
4. scd6∆edc3∆
8. scd6∆edc3∆
