# Supplementary material for: Decapping activators Edc3 and Scd6 act redundantly with Dhh1 in post-transcriptional repression of starvation-induced pathways
Source: eLife. 2025 Nov 25;13:RP102287. doi: 10.7554/eLife.102287 (PMC12646578; doi:10.7554/eLife.102287)
Supplement: Figure 6—source data 2. [file elife-102287-fig6-data2.zip › Fig. 6B-source data 1. PPT file containing original blots indicating relevant bands_10-27-25.pptx]

## Slide 1
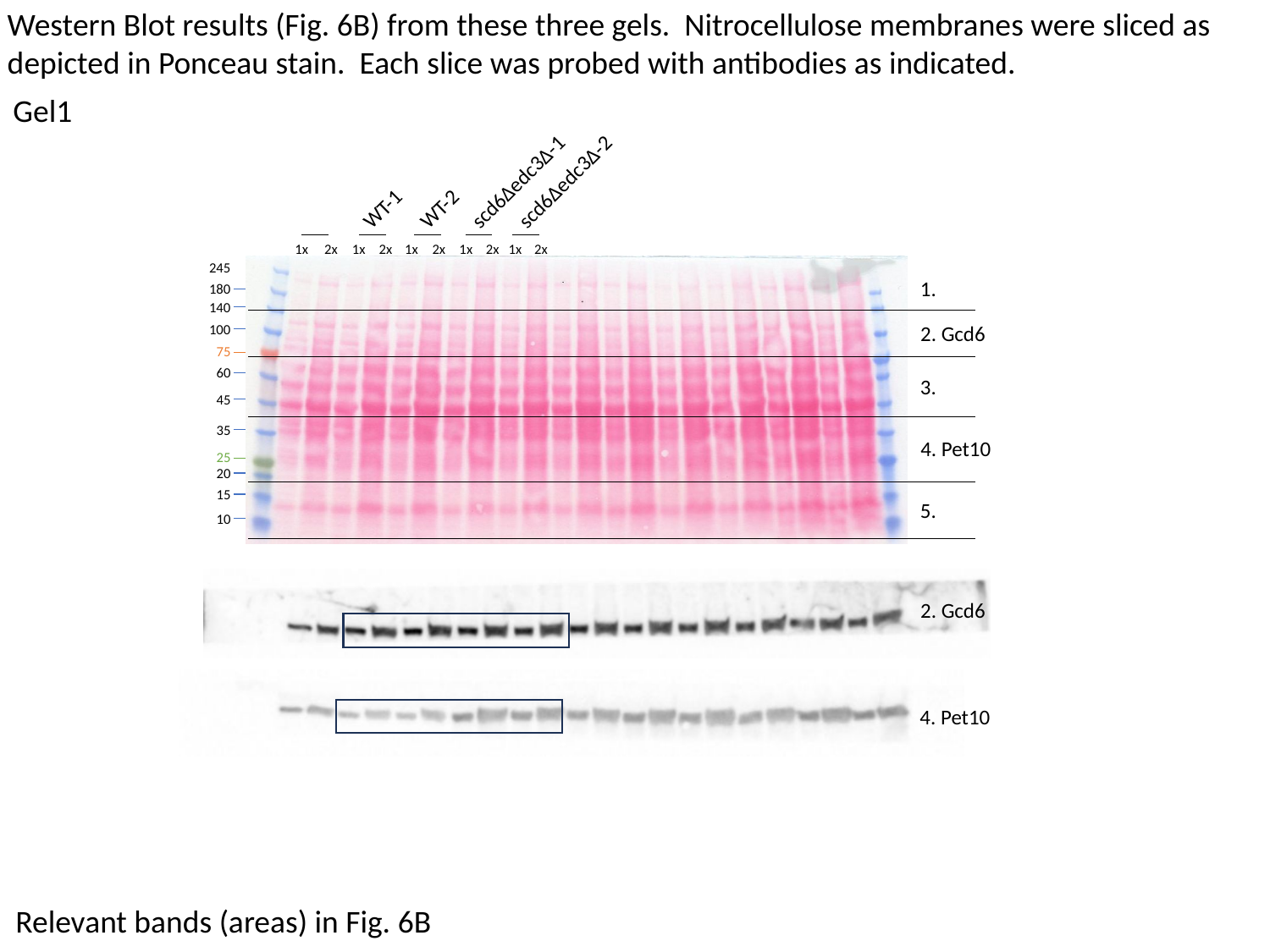

Western Blot results (Fig. 6B) from these three gels. Nitrocellulose membranes were sliced as depicted in Ponceau stain. Each slice was probed with antibodies as indicated.
Gel1
scd6∆edc3∆-1
scd6∆edc3∆-2
WT-1
WT-2
1x
2x
1x
2x
1x
2x
1x
2x
1x
2x
245
1.
180
140
2. Gcd6
100
75
60
3.
45
35
4. Pet10
25
20
15
5.
10
2. Gcd6
4. Pet10
Relevant bands (areas) in Fig. 6B

## Slide 2
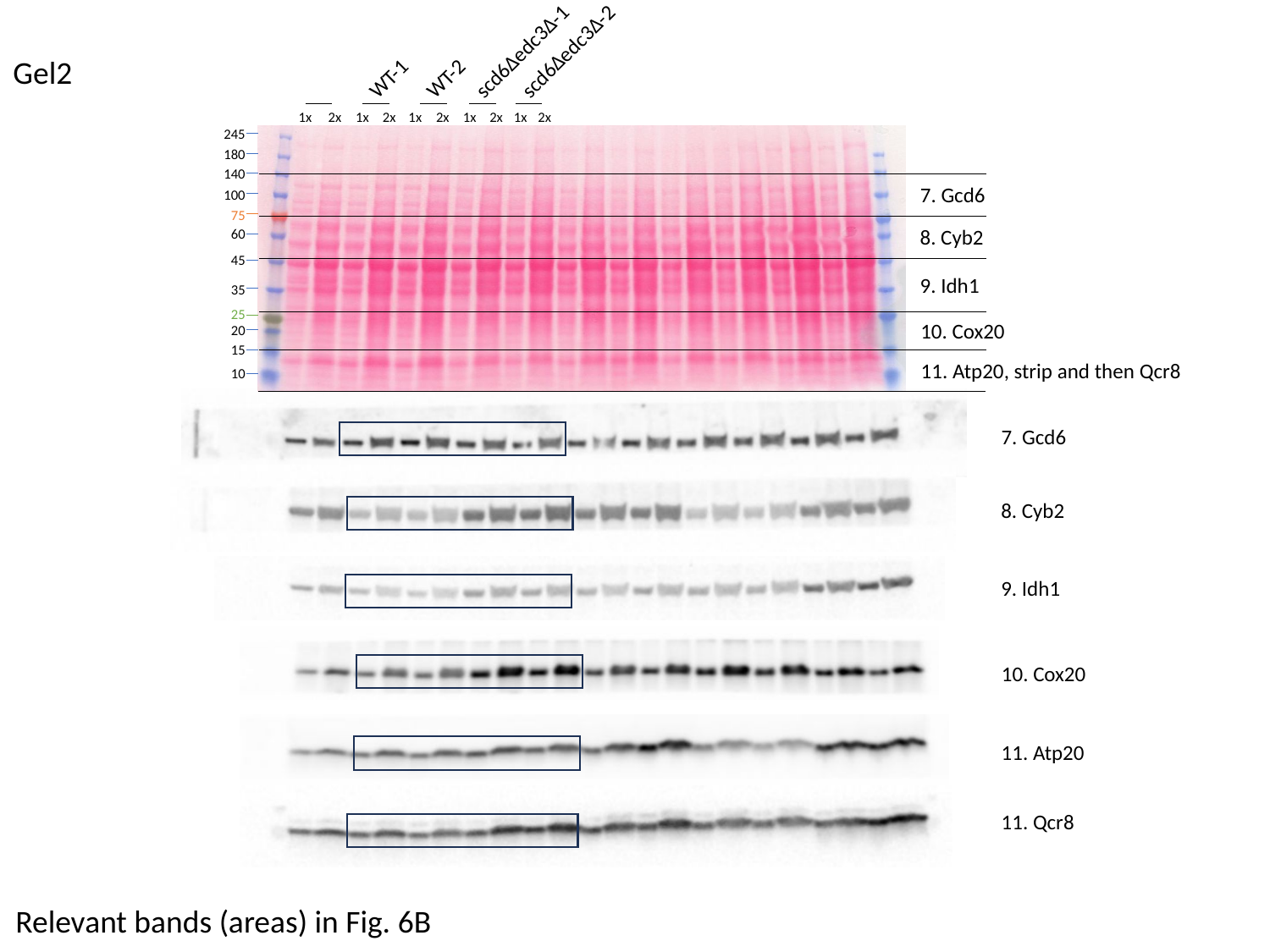

scd6∆edc3∆-1
scd6∆edc3∆-2
Gel2
WT-1
WT-2
1x
2x
1x
2x
1x
2x
1x
2x
1x
2x
245
180
140
100
75
60
45
35
25
20
15
10
7. Gcd6
8. Cyb2
9. Idh1
10. Cox20
11. Atp20, strip and then Qcr8
7. Gcd6
8. Cyb2
9. Idh1
10. Cox20
11. Atp20
11. Qcr8
Relevant bands (areas) in Fig. 6B

## Slide 3
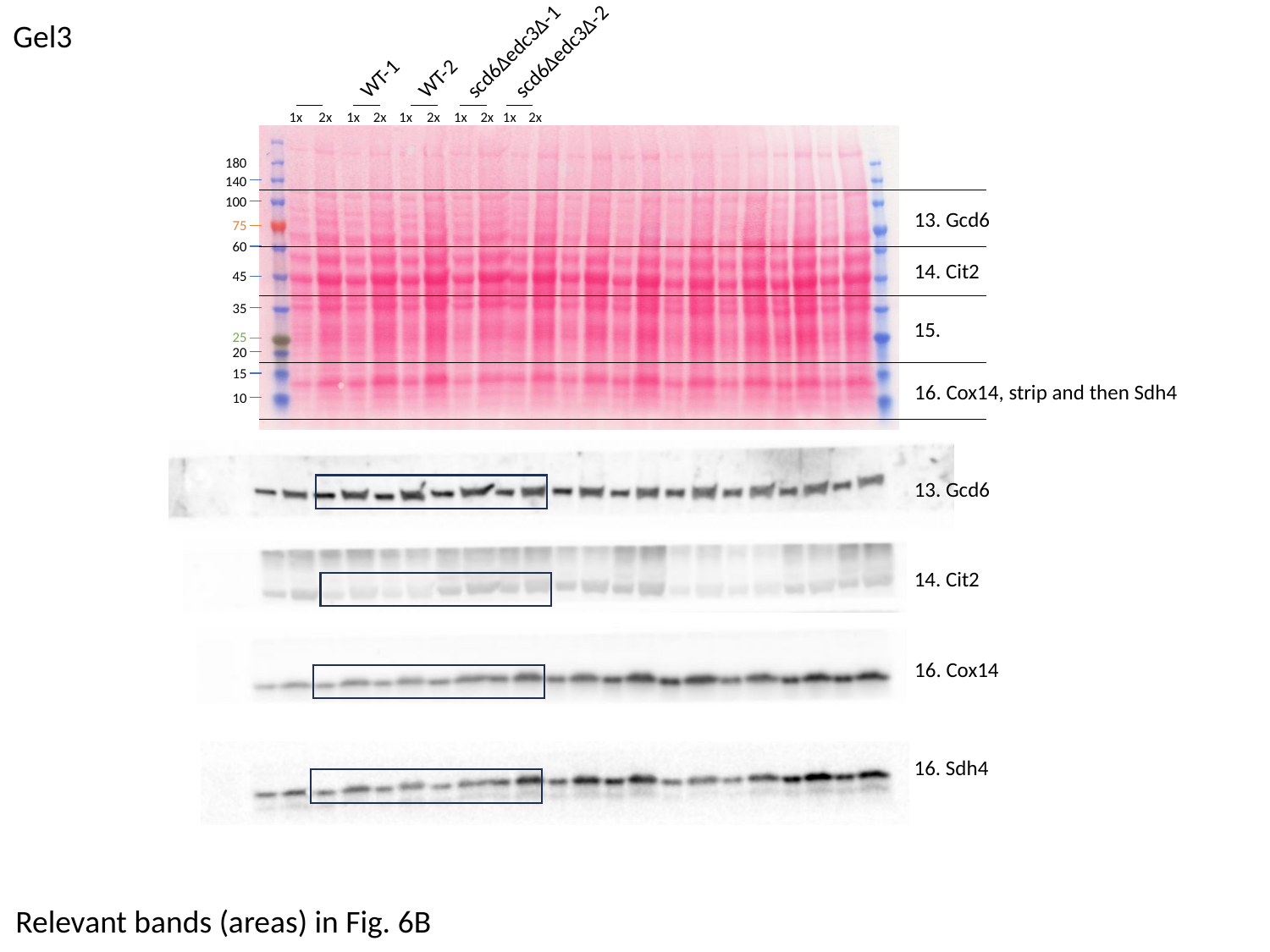

Gel3
scd6∆edc3∆-1
scd6∆edc3∆-2
WT-1
WT-2
1x
2x
1x
2x
1x
2x
1x
2x
1x
2x
180
140
100
13. Gcd6
75
60
14. Cit2
45
35
15.
25
20
15
16. Cox14, strip and then Sdh4
10
13. Gcd6
14. Cit2
16. Cox14
16. Sdh4
Relevant bands (areas) in Fig. 6B
